# Low-density lipoprotein receptor–related protein 5 governs Wnt-mediated osteoarthritic cartilage destruction

**DOI:** 10.1186/ar4466

**Published:** 2014-01-31

**Authors:** Youngnim Shin, Yun Hyun Huh, Kieun Kim, Suyeon Kim, Ka Hyon Park, Jeong-Tae Koh, Jang-Soo Chun, Je-Hwang Ryu

**Affiliations:** 1 Cell Dynamics Research Center and School of Life Sciences, Gwangju Institute of Science and Technology, 123 Cheomdangwagi-ro, Buk-gu, Gwangju 500-712, Republic of Korea; 2 BioImaging and Cell Dynamics Research Center, Gwangju Institute of Science and Technology, 123 Cheomdangwagi-ro, Buk-gu, Gwangju 500-712, Republic of Korea; 3 Department of Pharmacology and Dental Therapeutics, School of Dentistry, Chonnam National University, 77 Yongbong-ro, Buk-gu, Gwangju 500-757, Republic of Korea; 4 Research Center for Biomineralization Disorders, School of Dentistry, Chonnam National University, 77 Yongbong-ro, Buk-gu, Gwangju 500-757, Republic of Korea

## Abstract

**Introduction:**

Wnt ligands bind to low-density lipoprotein receptor–related protein (LRP) 5 or 6, triggering a cascade of downstream events that include β-catenin signaling. Here we explored the roles of LRP5 in interleukin 1β (IL-1β)- or Wnt-mediated osteoarthritic (OA) cartilage destruction in mice.

**Methods:**

The expression levels of LRP5, type II collagen, and catabolic factors were determined in mouse articular chondrocytes, human OA cartilage, and mouse experimental OA cartilage. Experimental OA in wild-type, *Lrp5* total knockout (*Lrp5*^-/-^) and chondrocyte-specific knockout (*Lrp5*^*fl/fl*^;*Col2a1-cre*) mice was caused by aging, destabilization of the medial meniscus (DMM), or intra-articular injection of collagenase. The role of LRP5 was confirmed *in vitro* by small interfering RNA–mediated knockdown of *Lrp5* or in *Lrp5*^-/-^ cells treated with IL-1β or Wnt proteins.

**Results:**

IL-1β treatment increased the expression of LRP5 (but not LRP6) via JNK and NF-κB signaling. LRP5 was upregulated in human and mouse OA cartilage, and *Lrp5* deficiency in mice inhibited cartilage destruction. Treatment with IL-1β or Wnt decreased the level of *Col2a1* and increased those of *Mmp3* or *Mmp13*, whereas *Lrp5* knockdown ameliorated these effects. In addition, we found that the functions of LRP5 in arthritic cartilage were subject to transcriptional activation by β-catenin. Moreover, *Lrp5*^-/-^ and *Lrp5*^*fl/fl*^;*Col2a1-cre* mice exhibited decreased cartilage destruction (and related changes in gene expression) in response to experimental OA.

**Conclusions:**

Our findings indicate that LRP5 (but not LRP6) plays an essential role in Wnt/β-catenin-signaling-mediated OA cartilage destruction in part by regulating the expression levels of type II collagen, MMP3, and MMP13.

## Introduction

Osteoarthritis (OA), which is the most common chronic degenerative joint disorder worldwide, is characterized primarily by cartilage degradation and narrowing of the joint spaces [1]. Both genetic and acquired factors, such as obesity, mechanical influences and age, are involved in the complex pathogenesis of OA, whereby cartilage homeostasis is disrupted by biophysical factors (for example, mechanical stress) and biochemical factors (for example, proinflammatory cytokines). The chondrocyte is a unique resident cell that synthesizes cartilage-specific extracellular matrix (ECM) components as well as various catabolic and anabolic factors. The pathogenesis of OA activates various biochemical pathways in chondrocytes, leading to proinflammatory cytokine production, inflammation, degradation of the ECM by matrix metalloproteinases (MMPs) and a disintegrin and metalloproteinase with thrombospondin motifs (ADAMTS), and cessation of ECM synthesis via the dedifferentiation and apoptosis of chondrocytes [2,3]. However, the molecular mechanisms underlying OA are not yet fully understood. The elucidation of such mechanisms could facilitate the development of new and effective therapeutic targets for the treatment of OA.

The Wnt signaling pathway is involved in cartilage development and homeostasis, as evidenced by the fact that a number of Wnt proteins and Frizzled (Fz) receptors are expressed in chondrocytes [4] and the synovial tissues of arthritic cartilage [5]. Interestingly, both chondrocyte-specific conditional activation and selective inhibition of β-catenin in mice have been shown to yield OA-like phenotypes, albeit via different mechanisms [6,7]. Several additional lines of evidence link Wnt/β-catenin signaling with OA, further supporting the notion that the Wnt/β-catenin pathway plays a role in the pathophysiology of cartilage [8-10].

Low-density lipoprotein receptor-related protein 5 (LRP5), which, together with LRP6, forms a distinct subfamily of LRPs), is a coreceptor for Wnt ligands, whereby the interaction of LRP5 with Axin initiates Wnt signaling by binding to members of the Fz receptor family [11]. LRP5 is one of the most intensively studied regulators of bone remodeling, largely because *Lrp5* loss-of-function mutations cause the autosomal recessive human disorder osteoporosis-pseudoglioma syndrome (OPPG) [12], whereas activating mutations in *Lrp5* cause high bone mass syndrome [13]. *Lrp6-*deficient mice display phenotypes similar to those seen in several *Wnt* knockouts (KOs) and die between embryonic day 14.5 and birth [14]. Despite the clear association of LRP5 with Wnt signaling and the involvement of Wnt/β-catenin signaling in cartilage degeneration, however, relatively few researchers have reported the involvement of LRP5 in OA pathogenesis. The OA susceptibility locus on chromosome 11q12-13 is in close proximity to the *Lrp5* gene, and a single polymorphism in *Lrp5* can confer increased risk for spinal OA and osteophyte formation [15]. LRP5 expression is increased in articular cartilage from OA patients and has been linked to increased MMP13 expression in chondrocytes [16]. Furthermore, bone morphogenetic protein 2–induced activation of Wnt/β-catenin signaling, which has been linked to enhanced catabolic activity of LRP5, contributes to hypertrophy in OA chondrocytes [17]. However, in a recent study, investigators reported that LRP5 deficiency could increase (rather than decrease) cartilage degradation in instability-induced OA [18]. Given this apparent discrepancy, additional work is clearly warranted to elucidate the molecular mechanisms underlying the LRP5-mediated regulation of OA pathogenesis.

In our present study, we investigated the distinct expression patterns of LRP5 and LRP6 in OA cartilage, elucidated the catabolic regulation of LRP5 in experimental OA using total and chondrocyte-specific conditional KO mice and examined the mechanisms underlying the LRP5-induced modulation of Wnt/β-catenin signaling. Our findings indicate that LRP5 (but not LRP6) plays an essential role in Wnt/β-catenin signaling–mediated OA cartilage destruction by upregulating catabolic factors (for example, MMP3 and MMP13) and downregulating the anabolic factor type II collagen.

## Methods

### Mice

Imprinting control region (ICR) mice were used for the chondrogenesis studies, and male C57BL/6, *Lrp5*^-/-^, *Lrp5*^*fl/fl*^;*Col2a1-cre*[19], STR/ort and CBA/CaCrl mice were used for the experimental OA studies. The *Lrp5*^-/-^ and *Lrp5*^*fl/fl*^ mice targeting exons 6 through 8 of *Lrp5* (kindly provided by Dr Gerard Karsenty of Columbia University (New York, NY, USA) [20]) were backcrossed against the C57BL/6J strain for eight generations. The *Col2a1-cre*-transgenic mice were obtained from The Jackson Laboratory (Bar Harbor, ME, USA) and backcrossed with *Lrp5*^*fl/fl*^ mice to generate chondrocyte-specific conditional KO mice (*Lrp5*^*fl/fl*^;*Col2a1-cre*). The genotyping primers for *Lrp5*^-/-^, *Lrp5*^*fl/fl*^ and *Col2a1-cre* were the same as those described previously [20]. The STR/ort and CBA/CaCrl mice were obtained from Harlan Laboratories (Indianapolis, IN, USA). All protocols were reviewed and approved by the Institutional Animal Care and Use Committee of Chonnam National University.

### Human arthritic cartilage and experimental osteoarthritis

Human OA cartilage was sourced from individuals undergoing arthroplasty [21]. Human cartilage was kindly provided by Dr Churl-Hong Chun of Wonkwang University (Iksan, Republic of Korea). The Institutional Review Board of the Wonkwang University Hospital approved the use of these materials, and all individuals provided written informed consent to be donors before undergoing surgery. Spontaneous OA in STR/ort mice [22] was examined at 28 weeks of age, with CBA/CaCrl mice used as controls. Aging studies were performed in 12-month-old mice, and experimental OA was induced in mice by destabilization of the medial meniscus (DMM) surgery [23] or by intra-articular injection of collagenase in 8-week-old male mice [24] and in in *Lrp5*^*-/-*^ mice and their wild-type (WT) littermates. Sham-operated and phosphate buffered saline–injected mice were used as controls for the DMM and collagenase-injected models, respectively. Mice were analyzed at 8 weeks after DMM surgery or 4 weeks after collagenase injection.

### Micromass culture and primary culture of articular chondrocytes

Mesenchymal cells were derived from the limb buds of ICR mouse embryos 11.5 days postcoitus and maintained as micromass cultures for induction of chondrogenesis as described previously [8]. Mouse articular chondrocytes were isolated from knee cartilage obtained from postnatal day 5 mice [25]. The articular cartilage was preincubated for 2 hours at 37°C with 0.2% trypsin and 0.2% type II collagenase and further digested with 0.2% type II collagenase for 90 minutes. On culture day 3, the cells were treated with recombinant interleukin 1β (IL-1β) (Calbiochem, San Diego, CA, USA), Wnt3a or Wnt7a (R&D Systems, Minneapolis, MN, USA) for 24 hours. Apoptosis was induced by treatment with an anti-Fas antibody (BD Biosciences, San Jose, CA, USA). Briefly, chondrocytes from articular cartilage of WT or *Lrp5*^*-/-*^ mice were incubated in the presence or absence of IL-1β (1 ng/ml) for 24 hours, then exposed to the anti-Fas antibody and recombinant protein G for an additional 6 hours. Hamster immunoglobulin G2 was used as a control. The cells were stained with fluorescein isothiocyanate–conjugated annexin V (BD Biosciences), and apoptotic chondrocytes were quantified by fluorescence-activated cell sorting analysis.

### Immunofluorescence microscopy and immunohistochemistry

Chondrocytes were cultured on glass coverslips, fixed with 3.5% paraformaldehyde and permeabilized with 0.1% Triton X-100. The cells were incubated for 1 hour with an antibody against type II collagen followed by incubation for 1 hour with an Alexa 488–conjugated secondary antibody (Invitrogen, Carlsbad, CA, USA). Ectopic expression of LRP5 was determined by labeling with an anti-LRP5 antibody and an Alexa 555–conjugated secondary antibody (Invitrogen). Apoptosis of chondrocytes in cartilage tissue was determined by terminal deoxynucleotidyl transferase deoxyuridine triphosphate nick end labeling (TUNEL) staining using a kit purchased from Roche Diagnostics (Indianapolis, IN, USA). Specimens were visualized under an IX81 inverted fluorescence microscope (Olympus America, Center Valley, PA, USA) driven by MetaMorph imaging software (Molecular Devices, Sunnyvale, CA, USA). Normal and OA human cartilage samples were frozen, sectioned at a thickness of 6 μm and subjected to Alcian blue and immunohistochemical staining. Mouse cartilage was fixed in 4% paraformaldehyde, decalcified in 0.5 M ethylenediaminetetraacetic acid (pH 7.4), embedded in paraffin and sectioned at a thickness of 6 μm. Cartilage destruction was evaluated by Safranin O staining and scored according to Mankin’s method [26]. Immunostaining of LRP5, MMP3, MMP13 and β-catenin in human and mouse cartilage was performed using standard techniques [21].

### RT-PCR and quantitative RT-PCR

Total RNA isolated from mouse articular chondrocytes and OA cartilage tissues was reverse-transcribed, and the resulting cDNA was PCR-amplified. The PCR primers and conditions used for mouse *Col2a1*, *Mmp3*, *Mmp13*, *Ptgs2*, *Nos2* and *Gapdh* were previously described [21]. The PCR primers for *Lrp5* and *Lrp6* were as follows: mouse *Lrp5*, sense: 5′-CTGAGGAACGTCAAAGCCATCAACTATG-3′, and antisense: 5′-TACTGGCTGTACGATGT TGGCATCTTC-3′; mouse *Lrp6*, sense: 5′-GCCCACTACTCCCTGAATGCTGACAAC-3′, and antisense: 5′-CCACTCCAACTGATCGTCCATCTAATC-3′; human *LRP5*, sense: 5′-GGGAGACGCCAAGACAGACAAGATCG-3′, and antisense: 5′-GGTGAAGACCAAGAAGG CCTCAGG-3′; and human *LRP6*, sense: 5′-ATTGTAGTTGGAG GCTTGGAGGATGC-3′, and antisense 5′-CCATCCATTCCAGCACGTTCTATC-3′. Quantitative RT-PCR (qRT-PCR) was performed using an iCycler (Bio-Rad Laboratories, Hercules, CA, USA) and SYBR *Premix Ex Taq* (Tli RNaseH Plus) (TaKaRa Bio, Kyoto, Japan).

### Western blot analysis

Total cell lysates were prepared with lysis buffer (pH 7.4) containing 150 mM NaCl, 1% Nonidet P-40, 50 mM Tris, 0.2% SDS, 5 mM NaF, a protease inhibitor cocktail and a phosphatase inhibitor cocktail. Proteins were resolved by SDS-PAGE, transferred to nitrocellulose membranes, detected by incubation with the appropriate primary antibody and a peroxidase-conjugated secondary antibody (Sigma-Aldrich, St Louis, MO, USA) and visualized using an enhanced chemiluminescence system (GE Healthcare Life Sciences, Pittsburgh, PA, USA). The primary antibodies used were purchased from ABGENT (LRP5, AP6157a; San Diego, CA, USA), EMD Millipore (type II collagen, MAB8887; Billerica, MA, USA), BD Biosciences (extracellular signal-regulated kinase (ERK), 610408; β-catenin, 610154), Santa Cruz Biotechnology (inhibitor of nuclear factor κB α, SC-371; and p38, SC-535; Santa Cruz, CA, USA) and Cell Signaling Technology (phosphorylated ERK, 9101; pp38, 9216; c-Jun N-terminal kinase (JNK), 9252; and phosphorylated JNK, 9255; Danvers, MA, USA).

### Transfection and reporter gene assay

Mouse articular chondrocytes were cultured for 3 days, transfected for 4 hours with *Lrp5* small interfering RNA (siRNA) (Dharmacon, Lafayette, CO, USA) or pSPORT6*-Lrp5* (Open Biosystems, Huntsville, AL, USA) using Lipofectamine 2000 reagent (Invitrogen), then treated with IL-1β, Wnt3a or Wnt7a. A nonsilencing control siRNA and empty vector were used as the negative controls. To determine the transcriptional activity of β-catenin-Tcf/Lef, we used a reporter gene assay. Chondrocytes were transfected with 1 μg of reporter gene (TOPflash) or control gene (FOPflash) (both from Upstate Biotechnology, Lake Placid, NY, USA) and 1 μg of pCMV*-*β-galactosidase using Lipofectamine 2000. The transfected cells were treated with IL-1β, Wnt3a or Wnt7a for 24 hours, then luciferase activity was measured and normalized with respect to transfection efficiency (as measured by β-galactosidase activity).

### Statistical analysis

The nonparametric Mann–Whitney *U* test was used to analyze data based on ordinal grading systems, such as International Cartilage Repair Society (ICRS) and Mankin scores. For qRT-PCR results and apoptotic cell numbers, the data were first tested for conformation to a normal distribution using the Shapiro-Wilk test, then analyzed by Student’s *t*-test (pairwise comparisons) or analysis of variance with *post hoc* tests (multiple comparisons) as appropriate. Significance was accepted at the 0.05 level of probability (*P* < 0.05).

## Results

### *Lrp5* (but not *Lrp6*) is upregulated via JNK and NF-κB pathways during IL-1β-mediated pathogenesis of chondrocytes

**Figure 1:**
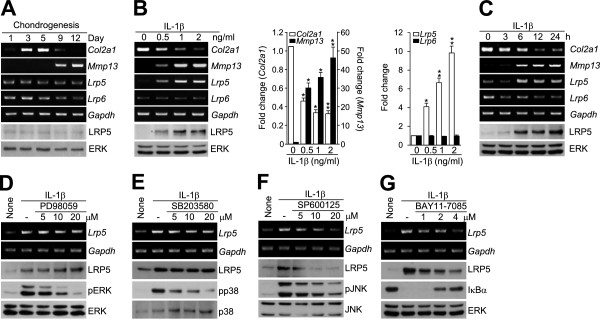
**Upregulation of LRP5 in interleukin 1β-treated mouse articular chondrocytes. (A)** Mesenchymal cells from mouse embryos were maintained as micromass cultures for 12 days, and the expression levels of *Col2a1*, *Mmp13*, *Lrp5*, *Lrp6* and *Gapdh* were detected by RT-PCR. LRP5 expression was confirmed by Western blot analysis. **(B)** and **(C)** Primary cultured mouse articular chondrocytes were treated with the indicated concentrations of interleukin 1β (IL-1β) for 24 hours (B) or with 1 ng/ml IL-1β for the indicated periods (C), and RT-PCR, quantitative RT-PCR and Western blot analysis were carried out. Values are presented as means ± SEM (*P < 0.001, **P < 0.0001; *n* = 6 independent experiments). **(D)** through **(G)** RT-PCR and Western blot analysis were performed on chondrocytes treated with IL-1β (1 ng/ml) for 24 hours in the presence of the indicated inhibitors (micromolar concentrations): PD98059, an extracellular signal-regulated kinase (ERK) inhibitor (D); SB203580, a p38 mitogen-activated protein (MAP) kinase inhibitor **(E)**; SP600125, a c-Jun N-terminal kinase (JNK) inhibitor **(F)**; and (2E)-3-[[4-(1,1-dimethylethyl)phenyl]sulfonyl]-2-propenenitrile (BAY11-7085), an inhibitor of nuclear factor κB α (IκBα) (G).

We first examined the expression levels of *Lrp5* and *Lrp6* during the chondrogenic differentiation of mesenchymal cells obtained from mouse embryonic limb buds and subjected to micromass culture. We found that *Col2a1* (which encodes the chondrocyte marker type II collagen) peaked on day 6 of micromass culture, *Lrp6* expression decreased beginning on day 6 and *Lrp5* expression was constant during chondrocyte differentiation (Figure 1A). The basal levels of *Lrp5* and *Lrp6* mRNA were very low in mouse articular chondrocytes. In pathogenic primary culture chondrocytes treated with IL-1β (the primary inflammatory cytokine involved in cartilage destruction), however, *Lrp5* expression was dramatically increased in a dose-dependent manner (Figure 1B) and a time-dependent manner (Figure 1C), whereas *Lrp6* expression was constant (Figures 1B and 1C). Consistent with our previous observations [20], IL-1β treatment increased the levels of *Mmp13* while abrogating *Col2a1* expression. Our qRT-PCR analysis revealed that IL-1β treatment triggered an approximately tenfold increase of *Lrp5* expression, but had no effect on *Lrp6* expression (Figures 1B and 1C). IL-1β treatment of chondrocytes triggered the activation of nuclear factor κB (NF-κB) and various mitogen-activated protein (MAP) kinase subtypes, including ERK, p38 kinase and JNK. Inhibition of ERK or p38 kinase had no effect on LRP5 expression (Figures 1D and 1E), but the blockade of JNK or NF-κB signaling (with SP600125 or BAY11-7085, respectively) markedly inhibited the IL-1β-induced increase in LRP5 expression (Figures 1F and 1G). These data indicate that LRP5 (but not LRP6) is increased during IL-1β-induced chondrocyte dedifferentiation and that this upregulation of LRP5 is mediated via the JNK and NF-κB signaling pathways.

### LRP5 expression is elevated in human and mouse osteoarthritic cartilage

**Figure 2:**
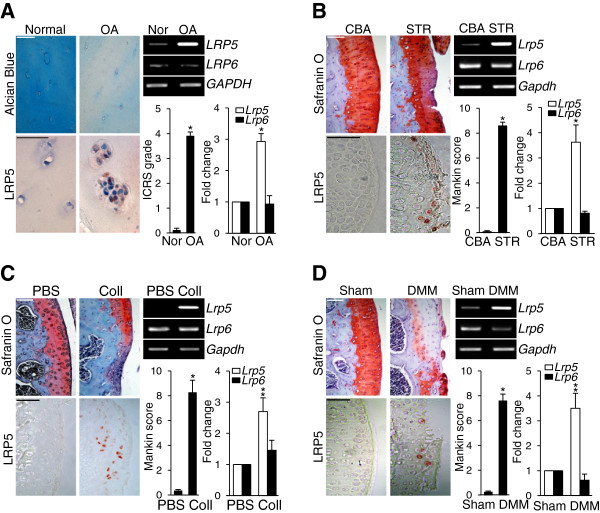
**LRP5 is upregulated in human and mouse experimental osteoarthritic cartilage. (A)** Undamaged (Normal/Nor) and damaged (osteoarthritis (OA)) human OA cartilage were examined with Alcian blue staining and immunostaining for LRP5. Six patients were independently assessed, and a typical result is presented. The mRNA levels of *Lrp5* and *Lrp6* in undamaged and damaged OA cartilage were examined by RT-PCR and measured by quantitative RT-PCR (qRT-PCR). **(B)** through **(D)** Safranin O staining and immunostaining for LRP5 were performed on cartilage samples from STR/ort (STR) OA mice and control CBA/CaCrl (CBA) mice (*n* = 8) **(B)**; C57BL/6 mice after intra-articular injection of collagenase (Coll) or control phosphate-buffered saline (*n* = 6) **(C)**; and C57BL/6 mice subjected to sham or destabilization of the medial meniscus (DMM) surgery (*n* = 10) (D). *Lrp5* and *Lrp6* mRNA levels were examined by RT-PCR and quantified by qRT-PCR (B) through **(D)**. Cartilage destruction was evaluated by International Cartilage Repair Society (ICRS) grade for human cartilage samples (A) and by Mankin score for mouse cartilage (B) through (D). Values are presented as means ± SEM (*P < 0.01, **P < 0.001; *n* > 7). Scale bar: 50 μm.

Because *Lrp5* expression was distinctly regulated during IL-1β-induced chondrocyte dedifferentiation, we examined whether LRP5 plays a role in OA cartilage destruction *in vivo*. We initially examined LRP5 levels in OA-affected human cartilage obtained from individuals who had undergone arthroplasty. The degree of cartilage damage in the human OA samples was ICRS grade 4 as confirmed by Alcian blue staining (Figure 2A). In these samples, LRP5 was significantly expressed in OA-affected human cartilage but barely detectable in normal cartilage (Figure 2A). This upregulation of *Lrp5* mRNA in human OA cartilage was confirmed by RT-PCR and qRT-PCR analyses (Figure 2A). We also found that the protein and mRNA levels of LRP5 were increased in cartilage from STR/ort mice (which were genetically predisposed to develop OA-like lesions in their medial tibial cartilage at age 6 months [21]) compared with that from control CBA/CaCrl mice (Figure 2B). We also observed increased LRP5 expression in mouse OA cartilage following collagenase injection (Figure 2C) and DMM surgery (Figure 2D). Thus, LRP5 expression was significantly elevated in all human and mouse OA cartilage samples examined in the present study.

### Catabolism-promoting gene regulation by LRP5 in dedifferentiated chondrocytes

**Figure 3:**
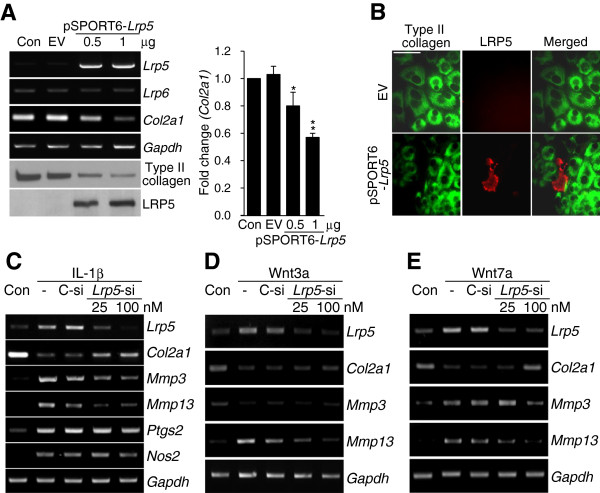
**Ectopic expression of LRP5 regulates type II collagen expression in mouse articular chondrocytes. (A)** Mouse articular chondrocytes were transfected with empty vector (EV; 1 μg) or the indicated amounts of pSPORT6-*Lrp5* for 24 hours. Downregulation of *Col2a1* expression in *Lrp5*-overexpressing chondrocytes was determined by RT-PCR and measured by quantitative RT-PCR. The protein levels of type II collagen and LRP5 were examined by Western blot analysis. Values are presented as means ± SEM (*P < 0.05 and **P < 0.01 versus EV-transfected cells; *n* = 8). **(B)** Articular chondrocytes cultured on glass coverslips were transfected with pSPORT6-*Lrp5* and immunostained with antibodies against type II collagen and LRP5. Scale bar: 20 μm. **(C)** through **(E)** Chondrocytes were transfected with 100 nM control small interfering RNA (C-si) or the indicated nanomolar concentrations of *Lrp5* siRNA (*Lrp5*-si) and treated with 1 ng/ml recombinant human IL-1β (C), 50 ng/ml recombinant mouse Wnt3a **(D)** or 500 ng/ml recombinant human Wnt7a (E). The mRNA expression levels of *Lrp5*, *Col2a1* and various catabolic factors (*Mmp3*, *Mmp13*, *Ptgs2* and *Nos2*) were assessed by RT-PCR.

Because the above-described results suggest that LRP5 may negatively regulate cartilage maintenance, we investigated the effects of LRP5 on catabolic and anabolic gene expression levels in chondrocytes. Ectopic expression of LRP5 significantly suppressed type II collagen expression at the transcript and protein levels (Figure 3A, left) but had no effect on the expression levels of catabolic genes such as *Mmp3*, *Mmp13*, *Adamts4*, *Adamts5* and *Ptgs2* (data not shown). Our qRT-PCR analysis clearly revealed that type II collagen expression was dose-dependently decreased by LRP5 overexpression (Figure 3A, right). Double-staining of type II collagen and LRP5 in primary articular chondrocyte cultures transfected with pSPORT-*Lrp5* indicated that cells highly expressing LRP5 were negative for type II collagen staining (Figure 3B). These data suggest that LRP5 expression was sufficient to cause chondrocyte dedifferentiation in our experimental system. Consistent with the unaltered expression of *Lrp6 in vitro*, however, LRP6 was barely detected in human and mouse OA cartilage samples, and LRP6 overexpression did not alter the expression levels of the tested genes (data not shown).

Next, we examined the effects of siRNA-mediated knockdown of *Lrp5* in dedifferentiated chondrocytes. IL-1β is known to trigger the expression of various catabolic factors (for example, MMP1, MMP3, MMP9, MMP12, MMP13, ADAMTS4, ADAMTS5, nitric oxide synthase 2 and prostaglandin-endoperoxide synthase 2) in primary cultures of articular chondrocytes [20]. Accordingly, we examined the possibility that LRP5 mediates the IL-1β-induced expression of these catabolic factors in chondrocytes. siRNA-induced knockdown of *Lrp5* was found to block the IL-1β-induced upregulation of *Mmp3* and *Mmp13*, as well as the IL-1β-induced downregulation of *Col2a1* (Figure 3C). To further confirm the effects of LRP5 on *Mmp3* and *Mmp13* expression in dedifferentiated chondrocytes, we stimulated the canonical Wnt pathway with recombinant Wnt3a and Wnt7a proteins. Both Wnt3a and Wnt7a induced chondrocyte dedifferentiation by suppressing *Col2a1* expression and concomitantly increased *Lrp5* expression (Figures 3D and 3E). However, Wnt3a and Wnt7a had differential effects on MMP expression. Wnt3a triggered the induction of *Mmp13* but not *Mmp3* (Figure 3D), whereas Wnt7a stimulated both *Mmp3* and *Mmp13* (Figure 3E).

### *Lrp5*-knockout mice show inhibition of experimental osteoarthritis-induced cartilage destruction

**Figure 4:**
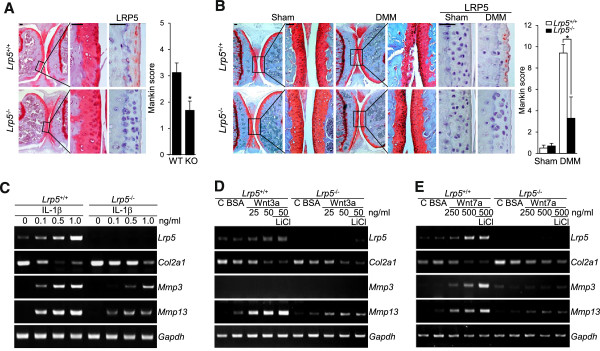
***Lrp5***-**knockout inhibits aging-induced and destabilization of the medial meniscus–induced osteoarthritic cartilage destruction. (A)** Spontaneous osteoarthritis (OA) in *Lrp5*-knockout (Lrp5-KO; *Lrp5*^*-/-*^) mice was examined at 12 months of age and compared with that in wild-type (WT) mice (*n* = 10 independent experimental animals). **(B)** Sham operation in the left knee joint and destabilization of the medial meniscus (DMM) surgery in the right knee joint were performed in WT and *Lrp5*^*-/-*^ mice (*n* = 20). Cartilage destruction was examined by Safranin O and hematoxylin staining of cartilage sections and measured by Mankin score. Values are expressed as means ± SEM (*P < 0.005). Scale bar: 50 μm. The presented immunostaining images of LRP5 in the knee articular cartilage are representative of at least seven independent tissue samples. **(C)** through **(E)** Chondrocytes obtained from WT and *Lrp5*^*-/-*^ mice were treated with the indicated concentrations (in nanograms per milliliter) of recombinant interleukin 1β (IL-1β) **(C)**, Wnt3a **(D)** or Wnt7a **(E)**, and the mRNA expression levels of *Lrp5*, *Col2a1*, *Mmp3* and *Mmp13* were assessed by RT-PCR. C: control, BSA: bovine serum albumin.

The specific *in vivo* functions of LRP5 were evaluated by inducing experimental OA in *Lrp5*^*-/-*^ mice via aging [21] or by DMM surgery [22]. Safranin O staining and Mankin score analysis revealed significant cartilage destruction in WT mice subjected to aging (Figure 4A) or DMM surgery (Figure 4B), whereas the degree of cartilage destruction was markedly reduced in *Lrp5*^*-/-*^ mice (Figures 4A and 4B). Consistent with our results following siRNA-mediated knockdown of *Lrp5* (Figures 3C through 3E), the IL-1β- or Wnt-mediated induction of *Mmp3* and *Mmp13* in articular chondrocytes obtained from *LRP5*^*-/-*^ mice were significantly decreased compared to those from their corresponding WT littermates (Figures 4C through 4E). To further determine whether the LRP5-mediated regulation of *Mmp3* and *Mmp13* expression occurred via the canonical Wnt/β-catenin signaling pathway, we examined the effects of LiCl treatment, which inhibits glycogen synthase kinase 3β (GSK3β). We found that LiCl treatment of chondrocytes from WT mice further enhanced the Wnt3a-mediated upregulation of *Mmp13* and the Wnt7a-mediated upregulation of *Mmp3* and *Mmp13*, whereas these parameters were unchanged in LiCl-treated *Lrp5*^*-/-*^ mice (Figures 4D and 4E).

### LRP5 potentiates Wnt/β-catenin signaling during osteoarthritis pathogenesis

**Figure 5:**
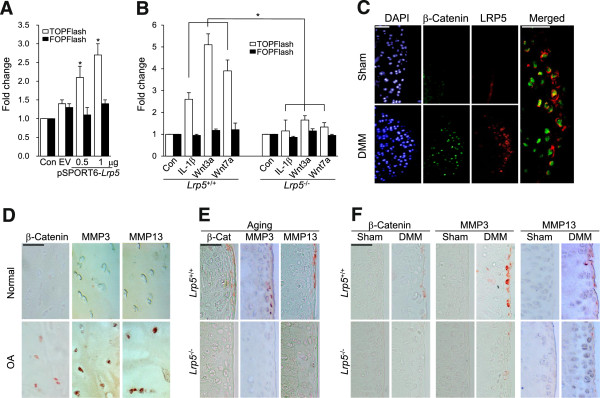
**Catabolic regulation of LRP5 mediates via β-catenin-Tcf/Lef signaling. (A)** Chondrocytes were transfected with 1 μg of empty vector (EV) or pSPORT6-*Lrp5* plus the TOPflash or FOPflash reporter constructs. After 24 hours, transcriptional activation by β-catenin was determined by luciferase reporter gene assays (*n* = 7 independent experiments). **(B)** Chondrocytes obtained from wild-type (WT) and *Lrp5*^*-/-*^ mice were treated with 1 ng/ml interleukin 1β (IL-1β), 50 ng/ml Wnt3a or 500 ng/ml Wnt7a, and transcriptional activation by β-catenin was evaluated by luciferase reporter gene assays (*n* = 6). Values are expressed as means ± SEM (*P < 0.005). **(C)** β-catenin and LRP5 expression levels in cartilage after sham operation or destabilization of the medial meniscus (DMM) surgery were determined by immunofluorescence microscopy. 4′,6-diamidino-2-phenylindole (DAPI) staining was used for visualization of nuclei. Scale bar: 20 μm. **(D)** β-catenin, matrix metalloproteinase 3 (MMP3) and MMP13 expression levels in undamaged (Normal) and damaged (osteoarthritis; OA) human osteoarthritic cartilage were examined by immunostaining. **(E)** and **(F)** β-catenin, MMP3 and MMP13 expression levels in spontaneous (aging-induced) osteoarthritic cartilage and DMM-induced osteoarthritic cartilage from *Lrp5*^*-/-*^ mice and their WT littermates were examined by immunohistochemistry. Scale bar: 50 μm.

Because GSK3β activity is primarily responsible for the degradation of β-catenin, we next examined whether the expression and/or activity levels of β-catenin could be regulated by LRP5. Ectopic expression of LRP5 in chondrocytes increased the transcriptional activation of β-catenin as determined by a Tcf/Lef reporter gene assay using TOPflash (containing an optimal Tcf/Lef-binding site) and FOPflash (containing a mutated Tcf/Lef-binding site) (Figure 5A). Treatment of chondrocytes from WT mice with IL-1β, Wnt3a or Wnt7a also increased the transcriptional activity of the β-catenin-Tcf/Lef complex, whereas this activity was completely blocked in cells from *Lrp5*^*-/-*^ mice (Figure 5B). Consistent with these observations, the expression levels of β-catenin and LRP5 were remarkably increased in OA cartilage induced by DMM surgery, and the β-catenin-expressing cells largely overlapped with the LRP5-expressing cells (Figure 5C). Moreover, the expression levels of β-catenin and MMP13 were increased in OA-affected human cartilage compared to healthy control cartilage (Figure 5D). Interestingly, the increases in β-catenin, MMP3 and MMP13 found in the OA cartilage of WT mice subjected to aging (Figure 5E) or DMM surgery (Figure 5F) were not observed in experimental OA cartilage samples from *Lrp5*^*-/-*^ mice.

**Figure 6:**
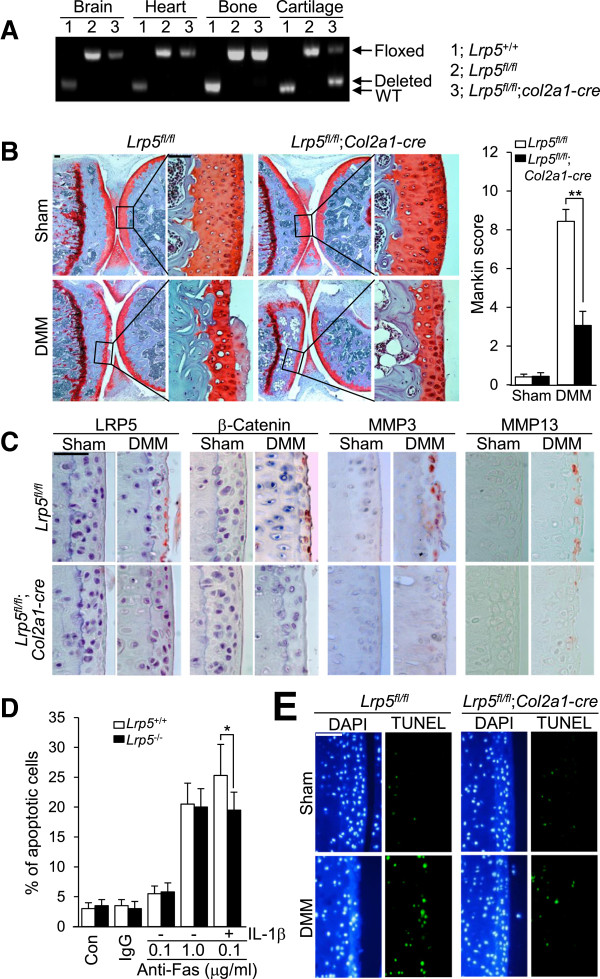
**Suppression of β-catenin and matrix metalloproteinase expression levels in chondrocyte-specific *Lrp5***-**knockout mice. (A)***Col2a1-Cre*-transgenic mice were backcrossed with *Lrp5*^*fl/fl*^ mice to generate *Lrp5*^*fl/fl*^;*Col2a1-Cre* mice. RT-PCR was used to examine deletion of the *Lrp5* gene in cartilage versus brain, heart and bone. WT: Wild type. **(B)** Cartilage destruction in *Lrp5*^*fl/fl*^;*Col2a1-cre* and control *LRP5*^*fl/fl*^ mice subjected to sham operations and destabilization of the medial meniscus (DMM) surgery was evaluated by Safranin O staining and Mankin score (*n* = 15 independent experimental animals). Scale bar: 50 μm. **(C)** The expression levels of LRP5, β-catenin, matrix metalloproteinase 3 (MMP3) and MMP13 in sham-operated control cartilage and DMM-operated osteoarthritic cartilage of *LRP5*^*fl/fl*^*;Col2a1-cre* and *LRP5*^*fl/fl*^ mice were determined by immunohistochemical staining. Scale bar: 50 μm. **(D)** Primary cultures of articular chondrocytes isolated from WT and *Lrp5*^*-/-*^ mice were treated with the indicated amounts of anti-Fas antibody for 6 hours or treated with 1 ng/ml IL-1β for 24 hours and with 0.1 μg/ml of anti-Fas antibody for an additional 6 hours. Apoptotic cells were quantified by fluorescence-activated cell sorting analysis (*n* = 5), Con: Control; IgG: Immunoglobulin G. **(E)** Apoptotic chondrocytes were visualized by 4′,6-diamidino-2-phenylindole (DAPI) staining and terminal deoxynucleotidyl transferase deoxyuridine triphosphate nick end labeling (TUNEL) assays of cartilage samples from *Lrp5*^*fl/fl*^;*Col2a1-cre* and control *LRP5*^*fl/fl*^ mice after sham operations and DMM surgery. Values are expressed as means ± SEM (*P < 0.05, **P < 0.001). Scale bar: 50 μm.

To control for unexpected effects from the lack of *Lrp5* in noncartilage tissues, we generated chondrocyte-specific conditional KO mice (*Lrp5*^*fl/fl*^;*Col2a1-cre*), whereby the *cre* recombinase gene specifically deleted the *Lrp5* gene from cartilage, but not other tissues, such as brain, heart and bone (Figure 6A). *Lrp5*^*fl/fl*^;*Col2a1-cre* and corresponding *Lrp5*^*fl/fl*^ control mice were subjected to induced OA by DMM surgery. Consistent with our data from the total KO mice, *Lrp5*^*fl/fl*^;*Col2a1*-*cre* mice exhibited significantly reduced cartilage destruction following DMM surgery compared with control *Lrp5*^*fl/fl*^ mice (Figure 6B) and did not show DMM surgery–induced upregulation of β-catenin, MMP3 and MMP13 expression levels in OA cartilage samples (Figure 6C)*.* We also examined whether the upregulation of LRP5 could potentiate chondrocyte apoptosis and found that chondrocyte apoptosis induced by 1 μg/ml anti-Fas antibody was not altered by *Lrp5* deficiency (Figure 6D). However, stimulation of apoptosis by IL-1β treatment in the presence of a low concentration (0.1 μg/ml) of anti-Fas antibody was slightly but significantly reduced in *Lrp5*-deficient chondrocytes (Figure 6D). As determined by TUNEL assay, apoptotic cells were also relatively reduced in DMM-induced OA cartilage from *Lrp5*^*fl/fl*^;*Col2a1*-*cre* mice compared to *Lrp5*^*fl/fl*^ mice (Figure 6E). Taken together, our results suggest that LRP5 induces chondrocyte dedifferentiation and promotes the expression of catabolic genes (for example, those encoding MMP3 and MMP13) by potentiating the Wnt/β-catenin signaling pathway.

## Discussion

Disturbance of cartilage homeostasis is a main cause of OA pathogenesis. In OA, cartilage destruction is initiated by defects in joint biomechanics in conjunction with predisposing factors (for example, age, genetics and various systemic aspects), leading to an imbalance of anabolic and catabolic factors [2]. Various biochemical pathways are modulated, resulting in the insufficient synthesis of cartilage matrix by chondrocytes, increased numbers of apoptotic chondrocytes [27] and degradation of the ECM due to increased production of MMPs and ADAMTS [2,3]. In this study, we demonstrate that *Lrp5* is a crucial catabolic regulator of Wnt/β-catenin signaling–mediated OA cartilage destruction. We first observed upregulation of LRP5 in human and experimental mouse OA cartilage samples. Our evaluation of the specific functions of LRP5 in OA pathogenesis further revealed that *Lrp5* deficiency in mice (*Lrp5*^*-/-*^) exerted a protective effect against OA pathogenesis. Our results additionally suggest that the catabolic regulation of LRP5 is associated with its capacity to initiate Wnt-mediated expression of catabolic factors, such as MMP3 and MMP13, and decrease the anabolic factor, type II collagen.

LRP5 and LRP6 are paralogs that are 70% identical, and both are capable of stimulating the Wnt/β-catenin signaling pathway. Even though they have redundant and overlapping functions [28,29], several previous reports have suggested that LRP5 and LRP6 also play distinct roles due to their differences in tissue distribution and ligand affinities [11,30]. For example, a loss-of-function mutation in *Lrp5* causes OPPG syndrome, a disorder involving low bone mass [12], whereas *Lrp6* deficiency (*Lrp6*^*-/-*^) in mice is an embryonic lethal disorder [14], and a heterozygous loss-of-function mutation in *Lrp6* (*Lrp6*^*+/-*^) is associated with decreased β-catenin signaling within articular cartilage and increased degenerative joint disease after ligament and meniscus injury [31]. These previous findings indicate that the specific receptors for LRP5 and LRP6 control different functions, presumably by interacting with distinct ligands of the Wnt family. In an effort to further confirm the catabolic regulation of *Lrp5*, we examined the expression levels of *Lrp5* and *Lrp6* in differentiating chondrocytes, human OA cartilage and cartilage samples from various experimental mouse models of OA. We observed distinct expression patterns for *Lrp5* and *Lrp6* during chondrogenesis and the IL-1β-induced dedifferentiation of chondrocytes. LRP5 expression in OA cartilage was increased, consistent with previous reports [15,16], whereas LRP6 expression was unaltered. These findings provide additional evidence that LRP5 and LRP6 have distinct expression patterns and may play different roles in OA cartilage destruction.

Previous studies have suggested that LRP5 may contribute to OA pathogenesis, but its function in OA cartilage destruction has been the subject of some controversy. LRP5 expression was found to be significantly upregulated in human OA cartilage [16], and a cohort study suggested that haplotypes of the *Lrp5* gene are risk factors for OA [15]. Conversely, however, mild instability-induced OA in *Lrp5*^*-/-*^ mice was reportedly associated with increased cartilage degradation [18]. Our data are inconsistent with the latter observation, even though the two studies seem consistent in terms of the method used to induce OA (DMM surgery), the duration after surgery (8 weeks) and the utilized mouse strain (C57BL6/J). To examine whether whole-body *Lrp5* deficiency could affect gene expression in other tissues by altering the susceptibility to pathogenic stimulation, we examined the chondrocyte-specific *in vivo* function of LRP5 in conditional KO mice (*Lrp5*^*fl/fl*^;*Col2a1-cre*) to exclude any unexpected side effects from the loss of *Lrp5* in other tissues. However, we found that the inhibitory effect of *Lrp5* deficiency on DMM surgery–induced OA cartilage degradation in *Lrp5*^*fl/fl*^;*Col2a1-cre* mice was consistent with the results from total *Lrp5*^*-/-*^ mice. These data indicate that LRP5 has catabolic effects during OA cartilage degradation.

In the current study, we used recombinant Wnt3a and Wnt7a as representative ligands of the canonical Wnt/β-catenin signaling pathway to evaluate the function of Lrp5. We did not examine the upregulation of Wnt molecules in the OA cartilage of our experimental systems, but Wnt3a is known to activate the canonical Wnt pathway and stimulate the expression of *Mmp13* and *Adamts4* in mouse chondrocytes [32,33]. We previously showed that IL-1β upregulates Wnt7a expression, thereby inhibiting type II collagen expression in chondrocytes [34]. Moreover, we found that the expression levels of various Wnt and Fz receptor isotypes were regulated by IL-1β [4]. In this study, we found that stimulation of canonical Wnt signaling via Wnt3a treatment caused upregulation of *Mmp13* in mouse articular chondrocytes, whereas Wnt7a treatment decreased *Col2a1* expression and increased *Mmp3* and *Mmp13* expression. Our observation that Wnt7a and IL-1β have similar effects on gene expression in chondrocytes is consistent with a previous report [4] in which we showed that IL-1β induced upregulation of *Wnt7a* in articular chondrocytes. Notably, however, the Wnt-mediated regulation of *Col2a1*, *Mmp3* and *Mmp13* were abrogated in primary cultured chondrocytes from *Lrp5*^*-/-*^ mice. On the basis of these data, we speculate that catabolic gene expression is convergently modulated by IL-1β in chondrocytes, with IL-1β-mediated *Wnt7a* and *Lrp5* expression triggering downregulation of *Col2a1* and upregulation of *Mmp3* and *Mmp13*, potentially contributing to the IL-1β-induced activation of β-catenin.

The catabolic effects of LRP5 may be attributable to its capacity to upregulate *Mmp3* and *Mmp13*, which encode proteins that are capable of degrading a variety of ECM components during the arthritic process [35]. Moreover, genetic studies in mice have clearly demonstrated that MMP3 and MMP13 play crucial roles in OA pathogenesis [36,37]. We observed that Wnt3a induced the expression of *Adamts4* (data not shown). Notably, however, *Adamts4* deficiency in mice did not show protective effects against OA cartilage destruction [38], whereas *Mmp13*-KO mice are resistant to OA cartilage erosion [37]. Therefore, the capacity of LRP5 to facilitate the Wnt-induced expression of MMP13 (a common catabolic factor that is regulated by both Wnt3a and Wnt7a) appears to be associated with the positive effects of LRP5 on OA cartilage destruction. The LRP5-induced downregulation of the anabolic factor type II collagen (a marker protein of chondrocytes) in articular chondrocytes also contributes to cartilage destruction. We found that ectopic expression of LRP5 induced the dedifferentiation of chondrocytes and was associated with the pathogenesis of OA. The apoptosis of chondrocytes, which is associated with the pathogenesis of OA, can be induced by a number of stimuli [39,40]. As we previously showed that Fas and its ligand are physiologically involved in chondrocyte apoptosis [41], in our present study we used an anti-Fas antibody to evaluate the role of LRP5 in chondrocyte apoptosis. The decreased chondrocyte apoptosis in *Lrp5*^*fl/fl*^;*Col2a1-cre* mice subjected to DMM surgery supports our contention that LRP5 plays a catabolic role in OA cartilage destruction.

## Conclusions

Herein we provide evidence suggesting that LRP5 is a catabolic regulator of OA pathogenesis and report that IL-1β treatment increases LRP5 expression largely via JNK and NF-κB signaling. On the basis of our results, we suggest that LRP5 plays a catabolic role in OA cartilage destruction by decreasing type II collagen synthesis (thus facilitating chondrocyte dedifferentiation), increasing MMP3 and/or MMP13 expression and promoting chondrocyte apoptosis. These results provide new insight into the mechanisms by which LRP5 upregulation contributes to OA cartilage and suggest that LRP5 could be a candidate therapeutic target for new strategies to treat or prevent OA.

## Abbreviations

ADAMTS: A disintegrin and metalloproteinase with thrombospondin motifs; DMM: Destabilization of the medial meniscus; ECM: Extracellular matrix; Fz: Frizzled; IL-1β: Interleukin 1β; JNK: c-Jun N-terminal kinase; LRP: Low-density lipoprotein receptor–related protein; MAP: Mitogen-activated protein; MMP: Matrix metalloproteinase; NF-κB: Nuclear factor κB; OA: Osteoarthritis; OPPG: Osteoporosis-pseudoglioma syndrome; qRT-PCR: Quantitative RT-PCR; TUNEL: Terminal deoxynucleotidyl transferase deoxyuridine triphosphate nick end labeling.

## Competing interests

The authors declare that they have no competing interests.

## Authors’ contributions

YS designed and performed most of the *in vitro* and *in vivo* studies. YHH conceived the project, performed histological evaluations and drafted the manuscript. KK carried out the immunoassays and performed the statistical analysis. SK and KHP participated in the animal studies and analyzed the data. JTK and JSC participated in the design of the study and helped to draft the manuscript. JHR conceived the project and is responsible for the overall design and oversight of the project. All authors read and approved the final version of the manuscript.
